# NHC-Stabilized Early Main Group Species: Reactivity and Emerging Catalysis

**DOI:** 10.1021/acscatal.5c08919

**Published:** 2026-02-27

**Authors:** Stuart Burnett, Scott Miller, Fáinché Murphy, Catherine E. Weetman

**Affiliations:** Department of Pure and Applied Chemistry, 3527 University of Strathclyde, 295 Cathedral Street, Glasgow G1 1XL, U.K.

**Keywords:** *N*-heterocyclic carbenes, main group, homogeneous catalysis, alkali metals, alkaline earth metals, group 13

## Abstract

*N*-Heterocyclic carbenes are commonplace in modern-day homogeneous catalysis due to their easily tunable steric and electronic features. Within the main group elements, NHCs have enabled the isolation of a range of complexes in unusual coordination environments and/or low oxidation states. With significant research efforts developing the transition-metal-like behavior of main group elements, emerging trends are now starting to showcase the potential of NHCs in enabling catalytic application of main group elements. This perspective highlights the potential of NHCs to transform main group catalysis, including a focus on relatively underexplored tethered and bis-NHC complexes for early main group elements.

## Graphical abstract

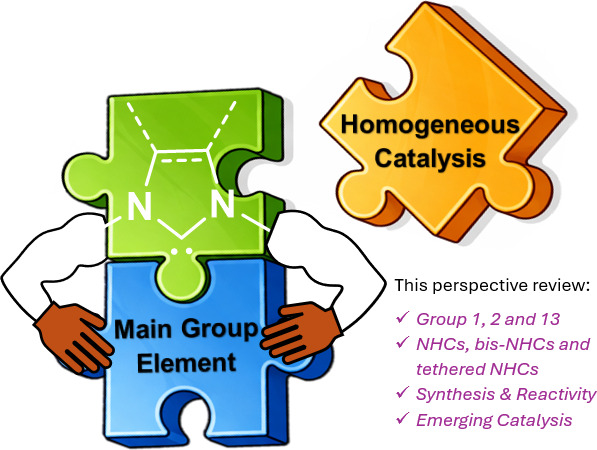

## 1 Introduction

Since their discovery by Arduengo et al. in 1991,^1^ *N*-heterocyclic carbenes (NHCs) have become established catalysts and supporting ligands for elements across the periodic table.^2–5^ The ease of synthesis and high degree of tunability have made these ligands highly favorable with applications in coordination chemistry, catalysis, materials, and medicinal chemistry. With the increasing demand for more sustainable transformations and with the rise of high technology, applications of NHCs are growing rapidly, and they are finding uses in quantum dots, biosensors, and optoelectronics due to their low cost, high efficiency, and recyclability.^6–8^ This perspective highlights the potential of NHCs to transform main group catalysis, including a focus on relatively underexplored tethered and bis-NHC complexes for early main group elements (groups 1, 2, and 13).

### 1.1 Bonding and Electronics

*N*-Heterocyclic carbenes are stable singlet carbenes that feature a neutral divalent carbon atom within a heterocyclic ring, which includes at least one nitrogen atom. Stable NHCs generally feature bulky *N*-substituents adjacent to the carbene carbon to kinetically stabilize the species by sterically disfavoring dimerization to the corresponding olefin (Wanzlick equilibrium).^9^ Classical NHCs such as IDipp (often abbreviated as IPr) (IDipp = 1,3-bis­(2,6-diisopropylphenyl)­imidazol-2-ylidiene) exhibit a singlet ground-state electronic configuration where the HOMO is a formally *sp* ^2^-hybridized lone pair, and the carbene carbon consists of *p*π acceptor character and is found in higher lying orbitals (LUMO + *n*) (Figure 1).

**Figure 1:**
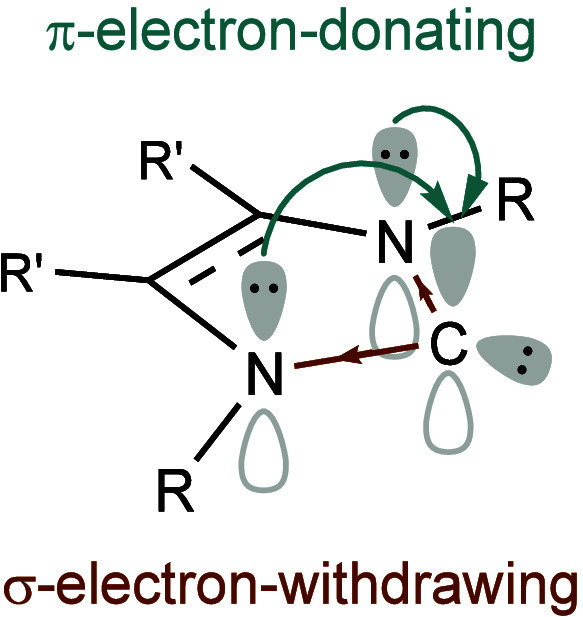
Electronic stabilizing effects in *N*-heterocyclic carbenes.

The adjacent nitrogen atoms are the most influential features of the heterocyclic ring as they stabilize the structure mesomerically by donating electron density into the empty carbon *p*-orbital and inductively by lowering the energy of the σ-type lone pair. As a result, the cyclic structure favors the singlet ground state as the carbene carbon is forced into a bent geometry. These factors determine the high stability of NHCs and their nucleophilic nature, resulting in strong σ-donating properties. Conversely, due to the presence of a low-lying C–N π* orbital that behaves as a formally vacant *p*-orbital on the carbon, NHCs also have negligible π-accepting abilities that can vary depending on the structure of the carbene.^10^

### 1.2 Classifications

One of the key reasons for the success of NHCs is the endless possibilities for structural and electronic modification, allowing for greater control of catalyst design. These modifications can be split into the following groups (Figure 2): backbone modification, *N*-substituents, and incorporation of a second heteroatom. All these possibilities have allowed for a wide variety of strong Lewis bases to be discovered each with different electronic and steric environments. The most widely researched basic structure is the unsaturated heterocycle with varying substituents on the nitrogen at the 1- and 3- positions, imidazol-2-ylidenes.

**Figure 2:**
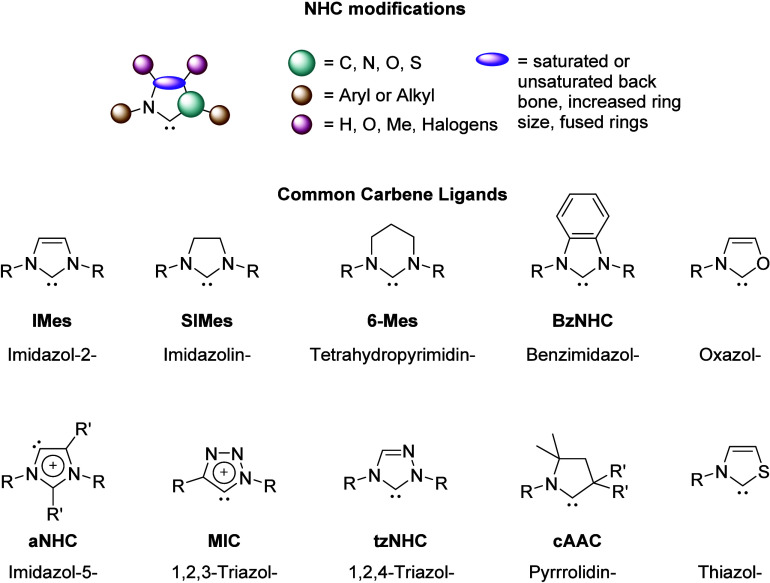
Classification of carbene ligands that contain at least one N atom in their heterocycle, along with the common abbreviation for when R = Mes (Mes = 2,4,6-trimethylphenyl) and IUPAC nomenclature. The suffix “ylidene” should be added to obtain the generic name of each NHC subclass.

While commonly used interchangeably in literature, for clarity in the context of this review, the abbreviations *a*NHC (carbenic carbon located on ring position 4 or 5) and MIC (ring containing three nitrogen atoms along with the carbenic carbon located on ring position 4 or 5) denote distinct variations of NHC ligands (see Figure 2 above). For the purpose of this review, the chalcogen-containing carbene ligands, along with cAAC’s, are included in Figure 2 only for reference and will not be discussed further.

Building on the basic imidazol-2-ylidene structure, further expansions in ligand design have led to the emergence of functionalized NHCs (Figure 3). Tethered NHCs are a developing class of ligands that contain all the desired features of NHCs but can also enable metal–ligand cooperativity in combination with electropositive metals. This is due to the use of “hard” electropositive metals in combination with “soft” NHC ligands; this results in an electronic mismatch and lability of the metal–NHC bond. The use of a tether, typically with an anionic donor group, essentially anchors the ligand to the electropositive metal and therefore keeps the NHC within close proximity to the metal center. This feature of tethered NHC ligands has drawn comparisons to “Frustrated Lewis Pair” (FLP) chemistry due to the donor–acceptor complexes that are formed and have been widely used in *f*-element and early transition metal organometallic chemistry.^4,11,12^

**Figure 3:**
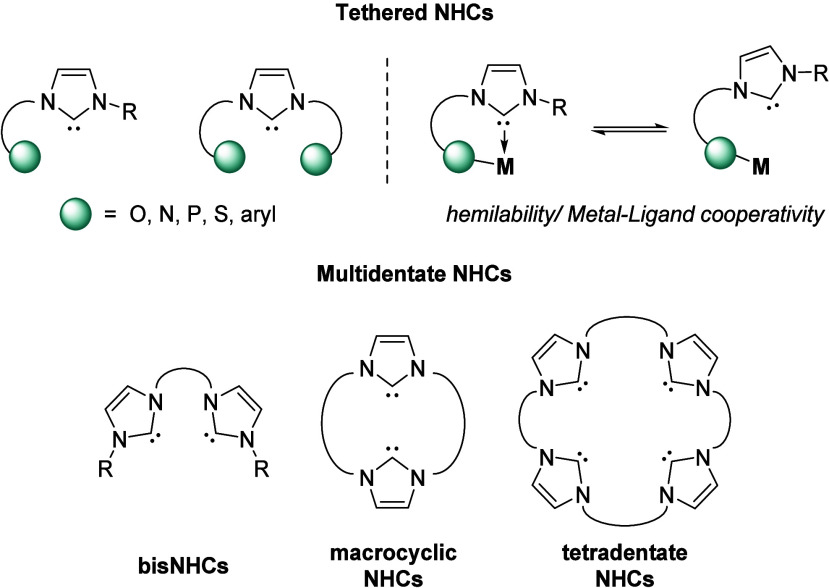
Functionalized NHC ligands based on the imidazole-2-ylidene structures.

Further functionality of the basic NHC ligand structure can be obtained through incorporation of additional NHC units within the ligand scaffold, resulting in multidentate NHC complexes. Use of a linker, typically an alkyl or aryl group, allows for easy synthetic modification and enables access to bi-, tri-, and tetradentate NHC ligands as well as incorporation into macrocyclic ligands (Figure 3). The use of functionalized NHC ligands also allows for extension to bimetallic and multimetallic coordination complexes.^12^

### 1.3 NHCs in Main Group Chemistry

NHCs have played a key role in the resurgence of main group chemistry; their strong σ-donating properties provide exceptional stability to highly reactive, electron-deficient main group centers.^13^ This stabilizing effect has facilitated the isolation of several unprecedented low-oxidation-state main group species, particularly in the field of main group multiple bonds, compounds containing elements in their zero oxidation states, and the isolation of main group cations and radicals (Figure 4).^14–19^

**Figure 4:**
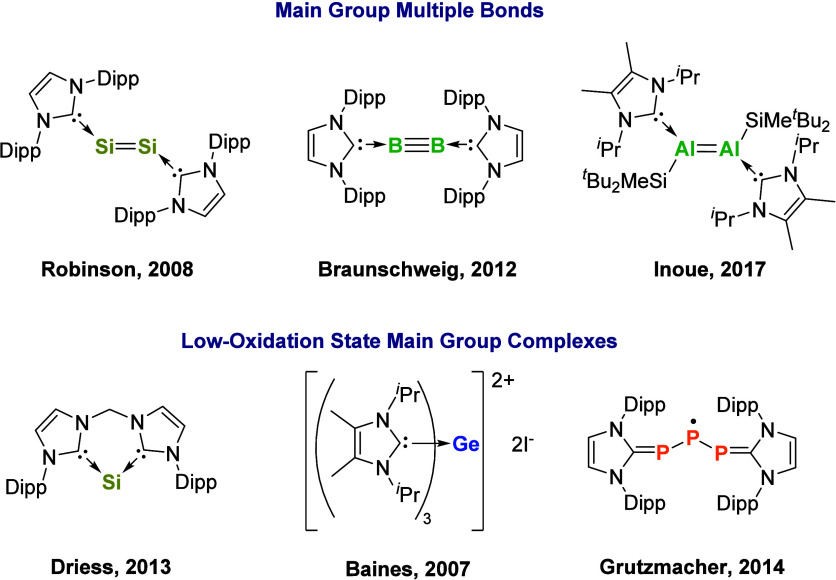
NHC stabilization of low-oxidation-state *p*-block compounds. Dipp = 2,6-diisopropylphenyl.

One of the driving forces behind the resurgence of *s*- and *p*-block chemistry was the discovery that main group elements in their lower oxidation states can behave as transition metal mimics in the activation of small molecules such as H_2_, CO_2_, and N_2_.^20,21^ This, combined with the high natural abundance, low cost, and often low toxicity of main group elements, makes research into molecular main group complexes more than just a fundamental curiosity but one that could provide a viable solution for the long-term sustainability of chemical industry.

Main group complexes have also shown themselves to be powerful catalysts, in some cases surpassing the activity of established transition metal systems. For example, the chemistry and reactivity of FLPs are now widely established and finding uses in a variety of industrial applications.^22–24^ While main group elements can access their lower oxidation states, the vast majority of main group catalytic cycles have been built around the stability of their higher oxidation state and are therefore redox inactive cycles.^25^ Catalytic cycles are thus built upon a series of σ-bond metathesis and insertion reactions, which allow for functionalization of a variety of substrates. These can be classified into two main categories: (i) protic cycles and (ii) hydridic cycles (Figure 5), which are determined by the polarity of the H-E reagent.

**Figure 5:**
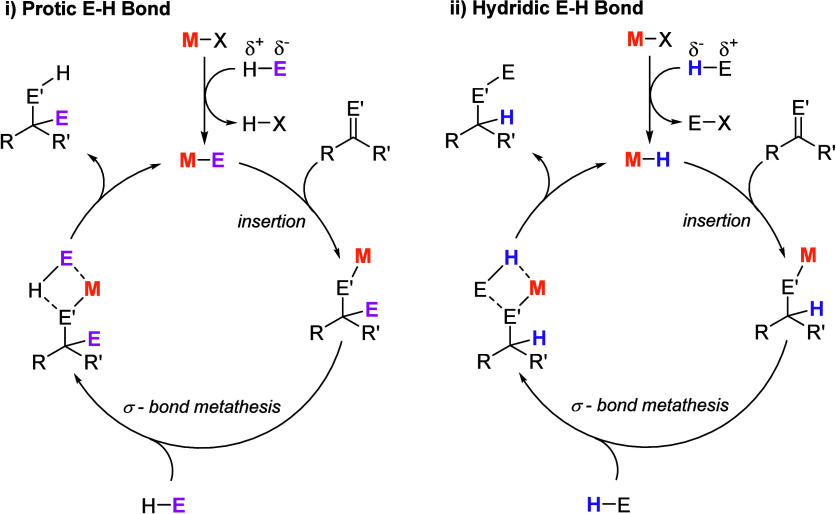
Protic (i) (e.g., E = N, O, P, S) and hydridic (ii) (e.g., E = B, Si) catalytic cycles assembled from a series of σ-bond metathesis and insertion reactions at metal centers. M = metal, E’ = N, O, S.

While the cycles highlighted in Figure 5 take advantage of the high stability of the common oxidation states of main group elements, it has been shown that it is possible to develop main group redox catalytic cycles akin to those of transition metals. Group 15 elements have recently demonstrated the ability to undergo traditional two-electron redox catalytic processes where turnover is achieved via a series of oxidative addition and reductive elimination steps. Examples of P­(III)/P­(V),^26,27^ Sb­(I)/Sb­(III),^28^ and Bi­(I)/Bi­(III)^29^ systems show that it is possible for *p*-block elements to behave as transition metal mimics in catalysis. For the rest of the *p-*block, the fundamental steps of oxidative addition and reductive elimination have been achieved, but the combination of these steps into catalytic turnover still represents a significant challenge, with only very recent examples of Al­(I)/Al­(III),^30^ Ga­(I)/Ga­(III),^31^ and Ge­(II)/Ge­(IV)^32^ redox processes appearing in the literature.

One of the key enabling features of group 15 redox catalysis was the ligand design; redox catalysis was observed when forcing the complexes into more planar geometries, away from their preferred pyramidal geometry via the use of pincer-type ligands.^33–35^ This is where NHC ligands can make an impact in main group catalysis. As highlighted earlier, these ligands can stabilize main group species in unusual geometries and lower oxidation states, and it is now a case of fine-tuning the balance of stability vs reactivity to enable catalytic transformations.

This perspective showcases the recent advances of NHCs in main group catalysis. Several recent reviews have comprehensively examined the synthesis and structural characteristics of NHC–main group complexes.^13,36^ As main group elements strive to establish themselves as viable alternatives to transition metals, we will highlight the recent progress in the use of the simple imidazol-2-ylidene NHC ligand scaffold, including bis- and tethered derivatives, and their role in the stabilization and catalytic application of early main group complexes (group 1, 2, and 13).

## 2 NHC-Stabilized Alkali Metal Complexes

Group 1 polar organometallic reagents are the foundation of synthetic organometallic chemistry; however, in comparison to the rest of the periodic table, the synthesis and reactivity of alkali metal NHCs are relatively underexplored as their prominent use is as carbene transfer agents. As such, the majority of advancements in this area have been meticulously detailed in the 2018 review of Inoue et al.^13^

Often overlooked, heavy alkali metal complexes are emerging as potential contenders in homogeneous catalysis and often outperforming their lighter congeners.^37^ However, due to the decreasing Lewis acidity and strength of metal–NHC bonds upon descending group 1, isolating heavy alkali metal NHC compounds is challenging and was elusive until recently. In 2025, Tamm and co-workers reported the first structurally characterized Rb and Cs NHC compounds (Scheme 1).^38^ Use of the group’s weakly coordinated fluoroborate anionic NHC (WCA-NHC) enabled the isolation of the series of group 1 alkali metal compounds (Na-Cs), with the Li complex previously being reported.^39^

**Scheme 1:**
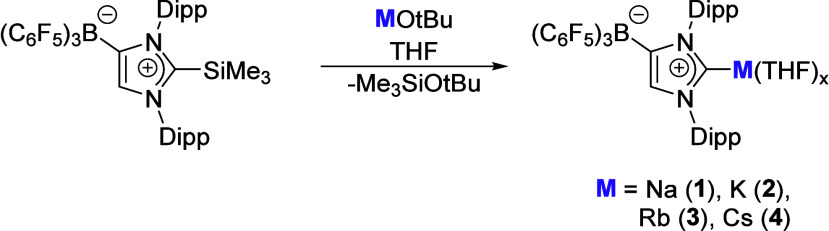
Synthesis of NHC Na-Cs Complexes **1**–**4**

In line with expectations, the metal–carbene bond lengths increase with the increasing ionic radius of the alkali metal cation (e.g., Na (**1**) 2.526(3) vs Cs (**4**) 3.3815(16) Å). Importantly and key to the stability of these complexes, secondary noncovalent interactions were found between the alkali metal and the Dipp substituents. This leads to a deviation in the yaw angle and is attributed to the preference for cation-π interactions with Rb and Cs, in line with other heavy alkali metal systems.^40–45^

Our group has similarly investigated the synthesis of such heavy alkali metal NHC complexes utilizing the fluorenyl-tethered NHC system (*vide infra*). Addition of the respective alkali metal amide reagent to *in situ* generated fluorenyl-NHC affords the anticipated Rb and Cs complexes **5** and **6**, respectively (Scheme 2).^46^ As is commonly observed in related heavy alkali metal systems, these complexes exhibit variable π-stabilization in the solid state between the metal cation and the fluorenyl anion. The first heterobimetallic Cs/Li NHC complex **7** could also be obtained either via treatment of the free carbene with a combination of Cs and Li amide bases or by the addition of the mixed metal base [CsLi­(N­(SiMe_3_)_2_)_2_] to benzene-*d*_6_ suspensions of **6.**

**Scheme 2:**
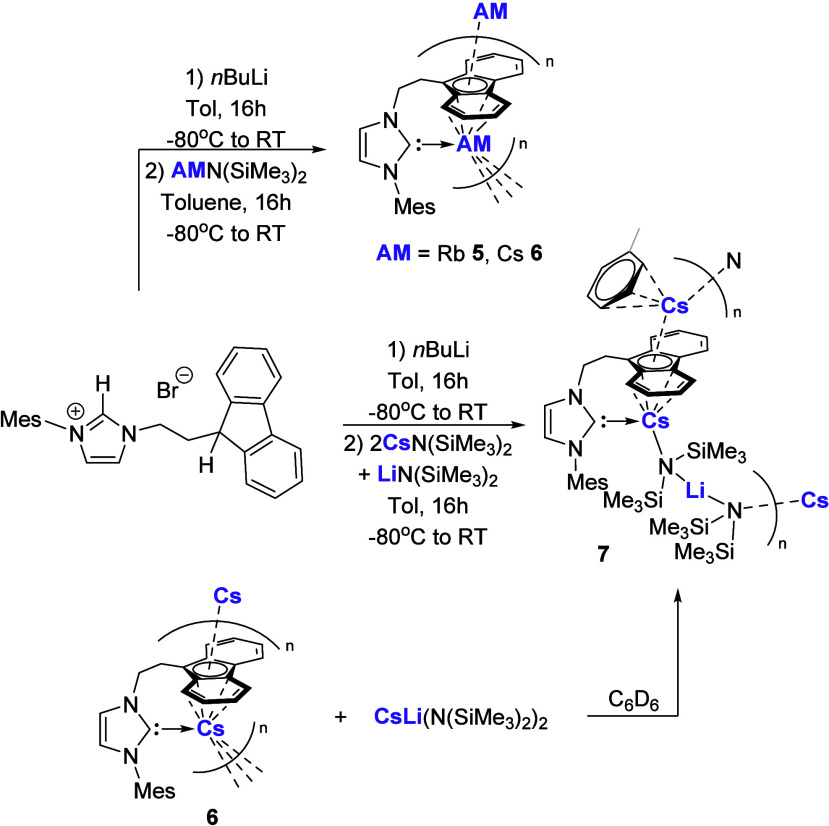
Synthesis of Homometallic, Fluorenyl-Tethered NHC Rb **5** and Cs **6** Complexes and Heterometallic Cs/Li Complex **7**

Since the first report of an anionic amido tethered NHC with group 1 metals in 2003 by Arnold and co-workers (Figure 6; **8**),^47^ several examples of alkali metal complexes have been realized featuring alkoxy,^48^ fluorenyl, and indenyl tethered NHCs^49–51^ (Figure 6; **9**–**11**, respectively). This additional tuneability within the ligand design has enabled the formation of bimetallic alkali metal coordinated NHC compounds that offer enhanced properties over their monometallic counterparts.

**Figure 6:**
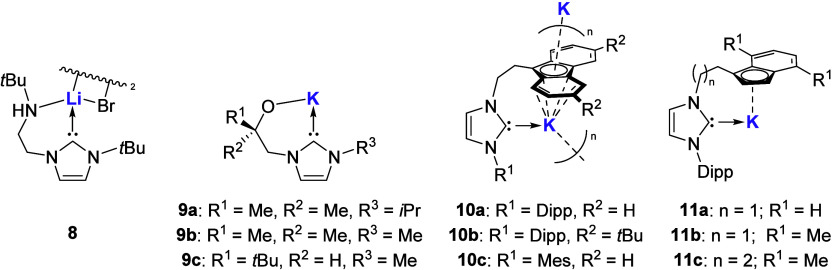
Examples of tethered alkali metal compounds.

In 2019, Evans and Mansell reported a series of homobimetallic fluorenyl-tethered NHC complexes (Scheme 3) with Li, Na, and K.^52^ Initial deprotonation of the saturated NHC tethered precursor (**12**) results in the formation of a spirocyclic carbene intermediate (**13**), which upon reaction with 1:1 mixtures of either LiPh/LiNR_2_ (R = SiMe_3_ or TMP (TMP = 2,2,6,6-tetramethylpiperidine)) or MCH_2_Ph/MN­(SiMe_3_)_2_ (M = Na, K) results in the homobimetallic complexes (Scheme 3; Li (**14**), Na (**15**), K (**16**)).

**Scheme 3:**
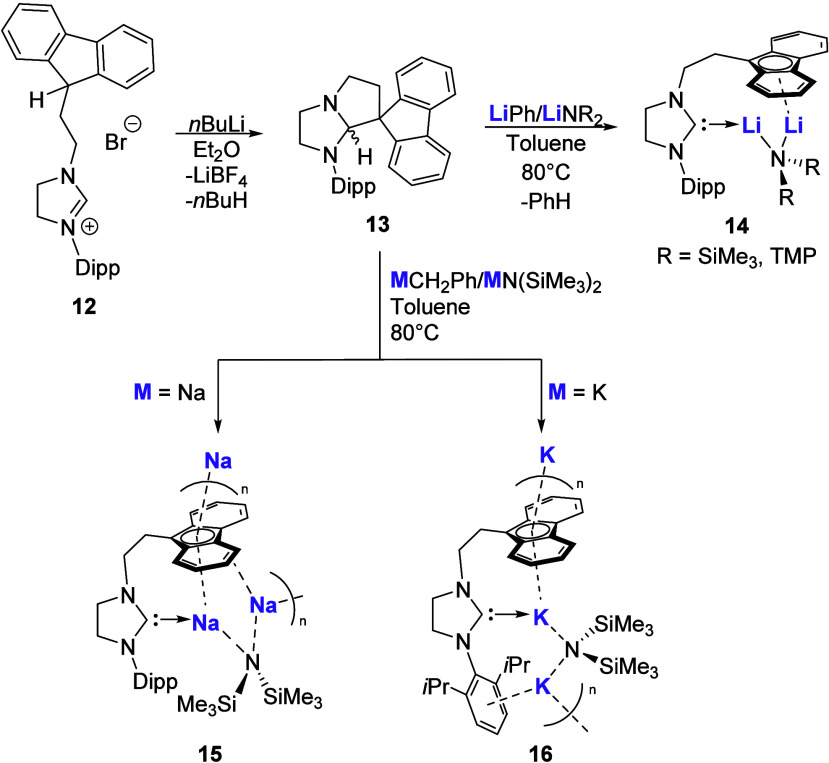
Synthesis of Alkali Metal Homobimetallic Fluorenyl-Tethered NHC Complexes

The Li (**14**) species was found to be soluble in aromatic solvents, while the polymeric Na (**15**) and K (**16**) exhibited poor solubility. All compounds (**14**–**16**) contain a bridging amide between the two metal centers; however, differences in the metal-arene coordination modes are observed across the alkali metal series with decreasing hapticity with increasing atomic number (η^6^ (Li), η^5^ (Na), and η^4^ (K)). This contrasts with what you would normally expect, as heavier alkali metal complexes typically prefer higher coordination numbers and show an increasing preference for π-arene interactions. Although several tethered alkali metal NHC complexes have been described, their applications have thus far been restricted to ligand transfer reagents, with no examples of either stoichiometric or catalytic reactivity reported.

Overall, reports of the bis-NHC ligand class with alkali metals are limited to a handful of examples. While modification of the alkyl linker between the two NHC units is possible, reported examples are exclusively the methylene bridge^53–55^ or borate^56^ bridge. The structural features of these bis-NHC alkali metal complexes have previously been covered,^13^ and similar to alkali metal tethered NHC complexes, no reactivity other than ligand transfer has been reported. However, it is important to note that two coordination modes—(a) chelating to single metal and (b) coordination to two metal centers—are possible for this ligand class (Figure 7).

**Figure 7:**
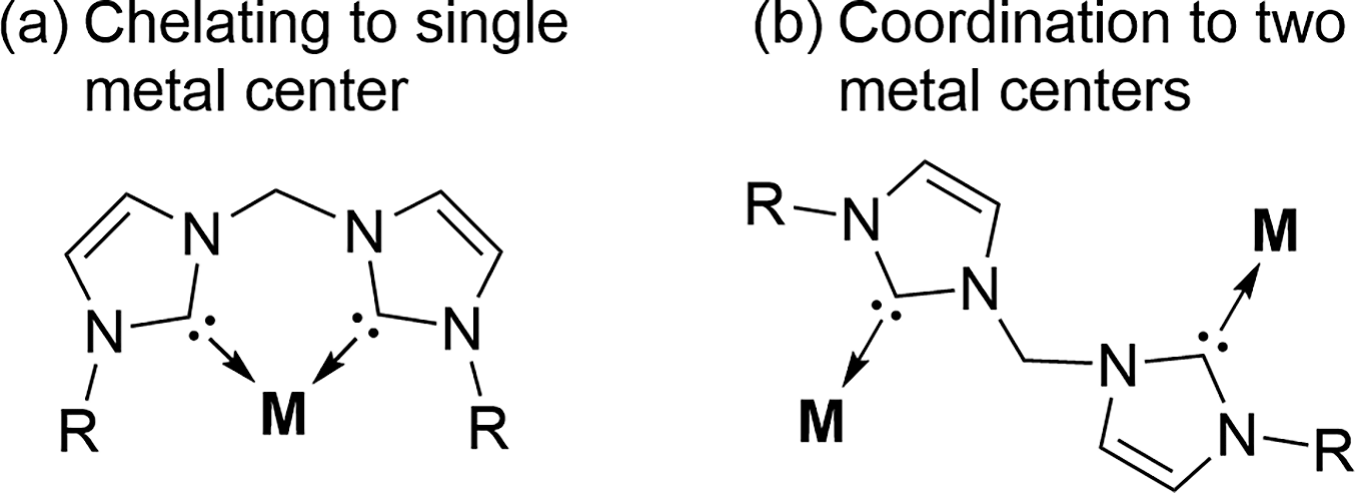
Two different coordination modes for bis-NHC complexes.

This adaptability in coordination modes can provide additional stability in catalytic reactions, as has been seen in transition-metal-based systems whereby the chelate effect has minimized catalyst decomposition pathways.^57–59^ Therefore, this ligand class has a wealth of untapped potential for alkali metal catalysis.

## 3 NHC Complexes of Alkaline Earth Metals

As is the case with the alkali metals, isolation and further reactivity studies of NHC-stabilized alkaline earth metal complexes are largely underdeveloped in comparison with the rest of the periodic table, with the majority of examples being confined to magnesium and calcium and with examples of their uses in catalysis limited to a select few cases.

### 3.1 NHC-Magnesium Complexes

The first NHC-magnesium adducts **17** and **18** were synthesized as far back as 1993 by Arduengo and co-workers via the addition of IAd (IAd = 1,3-bis­(1-adamantyl)­imidazol-2-ylidene) or IMes (IMes = 1,3-bis­(2,4,6-trimethylphenyl)­imidazol-2-ylidene) to diethylmagnesium (Scheme 4).^60^

**Scheme 4:**
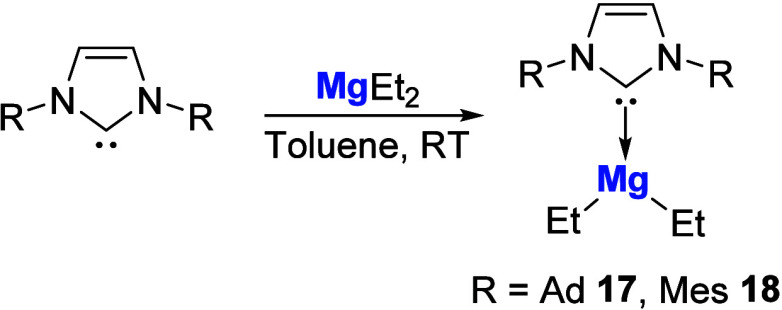
Synthesis of the First NHC-Mg Adducts by Arduengo and Co-workers

In 2009, Hill and co-workers reported the synthesis of an NHC-stabilized magnesium amide adduct, **19**, via the treatment of Mg­(N­(SiMe_3_)_2_)_2_ with 1 equiv of IDipp.^61^ Subsequent treatment of **19** with excess PhSiH_3_ at elevated temperatures afforded the NHC-stabilized magnesium hydride cluster **20** (Scheme 5) that features a Mg_4_H_6_ core coordinated by two NHCs and two N­(SiMe_3_)_2_ ligands. Interestingly, the cluster was found to be inert toward further Si-H/Mg-N metathesis reactions, highlighting its unusual stability.

**Scheme 5:**
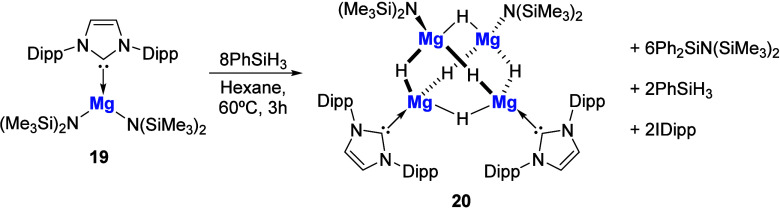
Synthesis of the Magnesium Hydride Cluster **20** by Hill and Co-workers

A related IMes-coordinated organomagnesium amide complex, **21**, reported by Nembenna and co-workers in 2017 was shown to be an effective precatalyst for the cross-dehydrocoupling of silanes with various primary and secondary amines.^62^ The mechanism proposed by the authors is shown in Scheme 6.

**Scheme 6:**
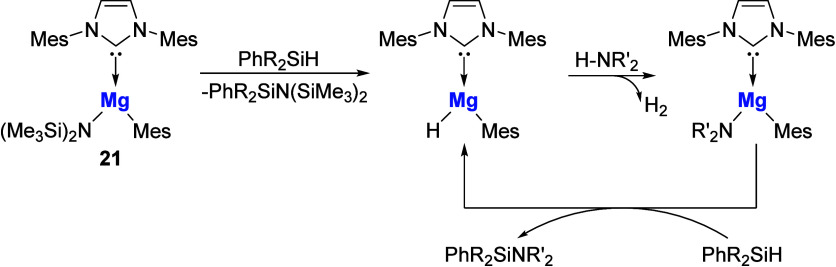
NHC-Mg Precatalyst in Cross-Dehydrocoupling of Silanes with Amines

Gilliard and co-workers in 2022 detailed a unique example of reversible migration of aminoborane within the coordination sphere of magnesium, a process typically only observed in transition metal chemistry.^63^ Through combined experimental and computational analysis, the authors demonstrated this dynamic process to result from the variable charge localization in the formed [NMe_2_BH_2_NMe_2_BH_3_]^−^ anion, along with the ability of NHCs to reversibly capture and release NMe_2_BH_2_ in the presence of Lewis acidic magnesium­(II) amides (Scheme 7). Such reversible “transition-metal-like” processes highlight the potential for even simple NHC-stabilized *s*-block metal complexes to participate in catalytic processes.

**Scheme 7:**
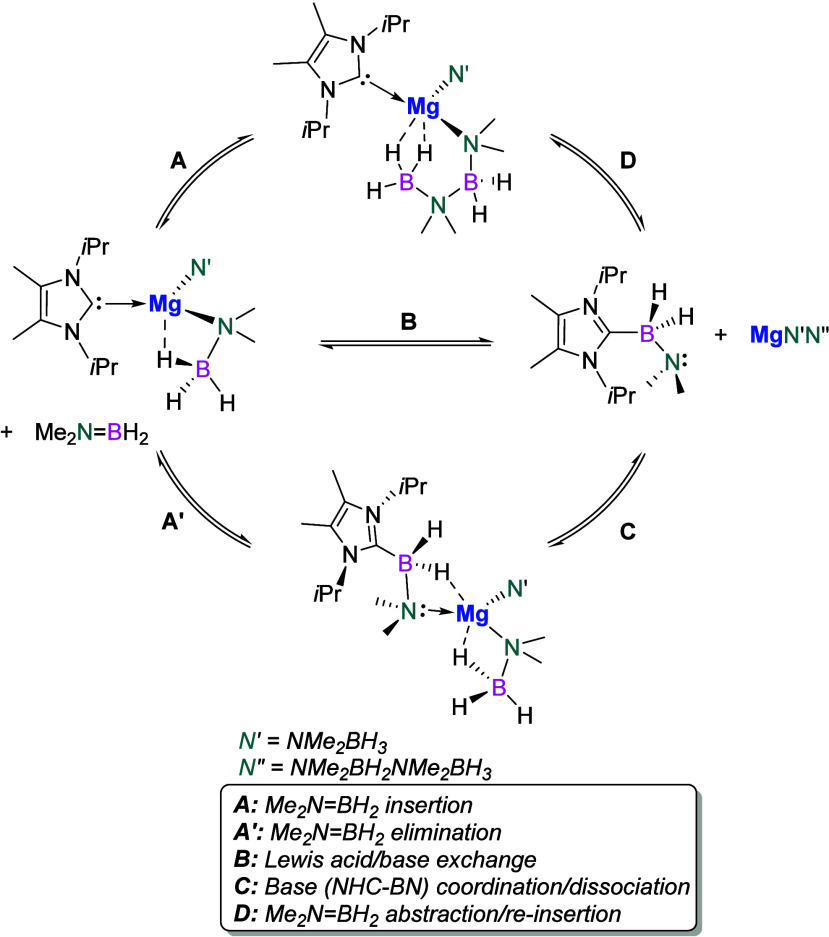
Mechanism for NHC-Mg Reversible Migratory Coupling of Aminoborane

### 3.2 NHC Complexes of the Heavy Alkaline Earth Metals

In 1997, Herrmann and Köcher detailed the isolation of the carbene adducts of calcium, strontium, and barium **22** and **23** through addition of 2 equiv of NHC (IMe (IMe = 1,3-dimethylimidazol-2-ylidene) or I^*t*^Bu (I^*t*^Bu = 1,3-di-*tert*-butylimidazol-2-ylidene)) to the respective metal amide (Scheme 8).^64^ A notable trend in solubility and thermal stability was observed upon descending the group. The calcium and strontium derivatives could be isolated at −36 °C, while the barium adducts were stable only in solution, precluding the acquisition of solid-state structural data, while also exhibiting fluxional dissociation and coordination in solution.

**Scheme 8:**
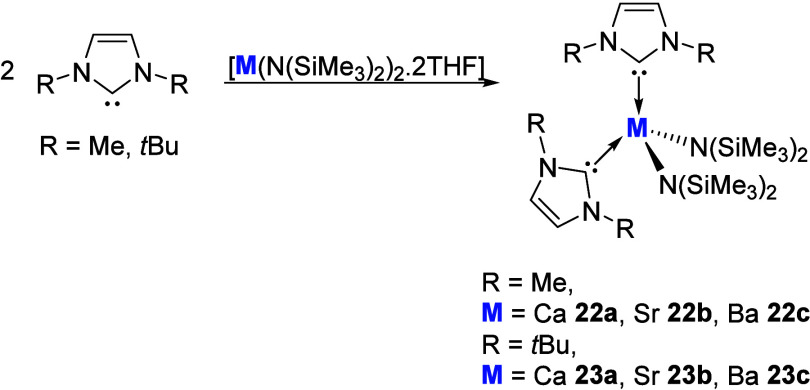
Synthesis of NHC Group 2 Amide Complexes Detailed by Herrmann and Köcher

Hill and co-workers subsequently reported in 2008 the synthesis of related mono-NHC-stabilized Ca, Sr, and Ba amide complexes via reaction of the imidazolium salt either with 1 equiv of Ca­(N­(SiMe_3_)_2_)_2_ to afford the chloride/amide adduct complex **24** or with 2 equiv of the respective metal amide to yield the bis-amide adduct species **25a**–**c** (Scheme 9).^65^ In an analogous fashion to their previously reported NHC-magnesium complex **19**, addition of free IDipp to Ca­(N­(SiMe_3_)_2_)_2_ as expected forms the corresponding [(IDipp)­Ca­(N­(SiMe_3_)_2_)_2_)] **26.**

**Scheme 9:**
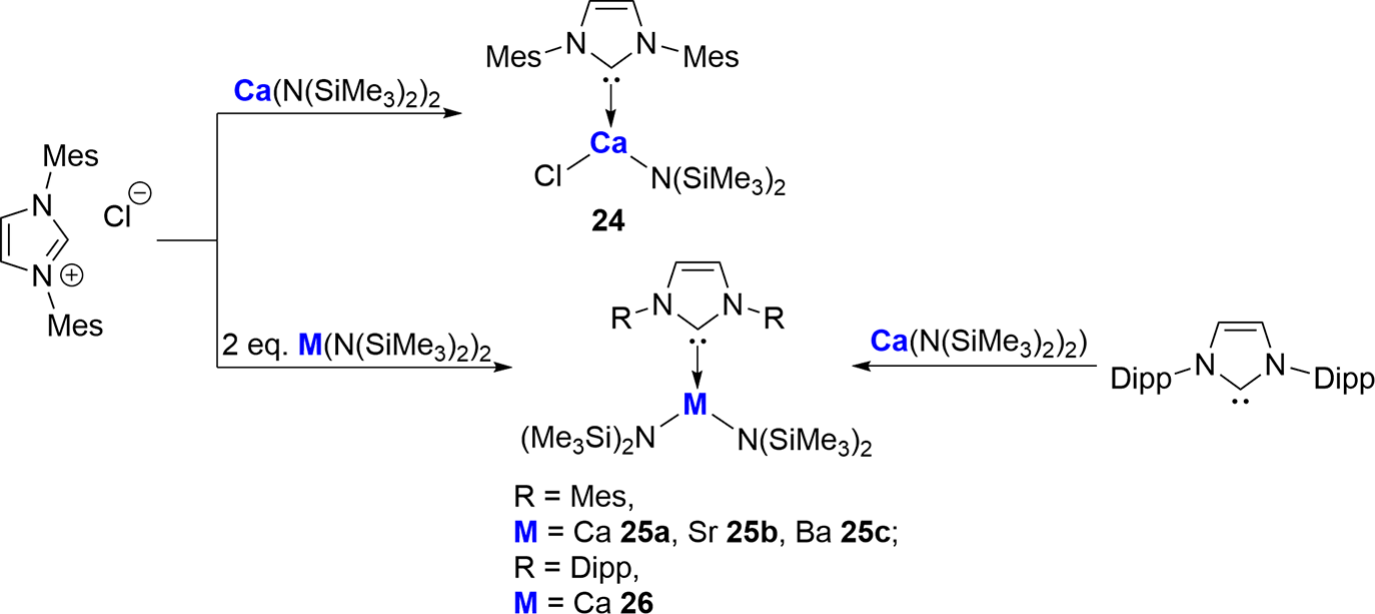
Hill and Co-workers’ NHC-Stabilized Ca, Sr, and Ba Complexes

Work by the Trifonov group demonstrated the catalytic potential of the NHC-calcium complex [(^Me^I*i*Pr)_2_Ca­(N­(SiMe_3_)_2_)_2_] (^Me^I*i*Pr = 1,3-di-*iso*-propyl-4,5-dimethyl-imidazolin-2-ylidene) in the addition of PH_3_ and PhPH_2_ to C–C double and triple bonds, which exhibits excellent regio- and chemoselectivity in the hydrophosphination of styrene.^66,67^ A related NHC-stabilized calcium dialkyl complex reported by Lin and Guan displayed similar high catalytic activity in the aforementioned cross-dehydrocoupling, while use of a chiral NHC ligand enables stereoselectivity of such calcium-mediated catalytic systems (Scheme 10).^68^

**Scheme 10:**
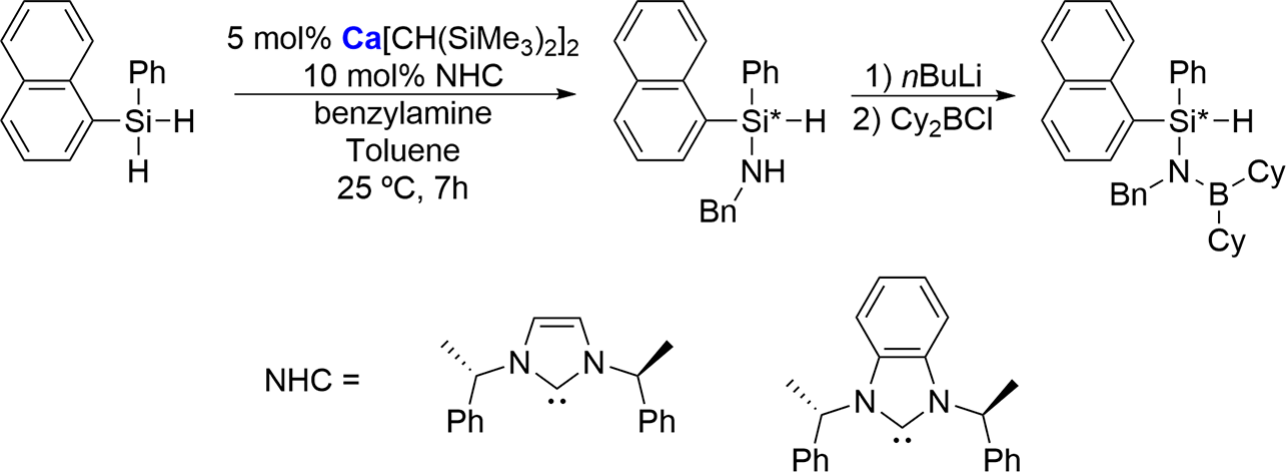
Cross-Dehydrocoupling of Silane and Benzylamine Using Chiral NHCs

### 3.3 Tethered NHC Ligands in Group 2 Chemistry

While the first report of tethered NHC alkaline earth metal complexes date back to 2004, in which the Arnold group detailed the synthesis of an amido-functionalized NHC-magnesium complex,^69^ subsequent work has been extremely limited, and again, only a select few examples of complexes in catalysis have been forthcoming. The same group in 2009 introduced a series of alkoxy-tethered NHC-magnesium complexes, which depending on the steric bulk of the *N*-substituent yielded either monomeric (R = Dipp **27**) or dimeric (R = *i*Pr **28**, Mes **29**) species (Scheme 11).^70^ Complexes **27** and **28** functioned as efficient catalysts in the ring-opening polymerization of *rac*-lactide, both at ambient temperature in the absence of an external initiator. The less sterically hindered **28** exhibited significantly higher catalytic activity, achieving 98% monomer conversion after 45 min in comparison to 27% for **27** within the same time scale. This rate difference was attributed to the bonding in these complexes. The strong Mg–O bond and concurrently weaker Mg–C_carbene_ bond suggest that the monomer insertion mechanism likely occurs between the Mg–C_carbene_ bond. The increased steric bulk around this in **27** hinders the accessibility of that Mg–C moiety and thus decreases the rate of polymerization.

**Scheme 11:**
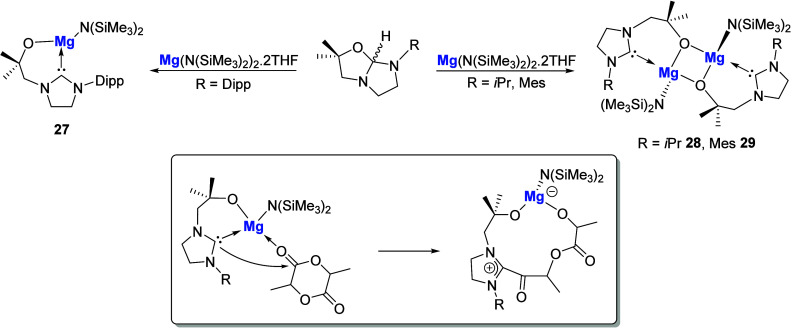
Synthesis of Monomeric and Dimeric Alkoxy-Tethered NHC-Mg Complexes

The Mukherjee group has very recently reported a series of Mg­(II), Ca­(II,) and Sr­(II) complexes stabilized by the fluorenyl-tethered NHC ligand (Scheme 12) either via treatment of the imidazolium salt proligand with the respective metal amide base followed by addition of K­(N­(SiMe_3_)_2_) to yield the corresponding NHC-metal amide complexes **31**–**33** or by the reaction of the potassium salt of the ligand with MeMgBr (complex **35**) or with MI_2_ (M = Ca, Sr) then K­(N­(SiMe_3_)_2_) (for **32** and **33**).^71,72^ Complexes **31**–**33** and **35** were screened as precatalysts in the hydroamination cyclization of aminoalkenes. Both Mg species proved highly effective, with **32** and **35** resulting in 99% substrate conversion within 30 min at ambient temperature. Interestingly, the Sr complex **33** showed no activity under the same conditions, the reason for which is suggested to be ligand exchange due to the potential instability of the intermediate species formed in the reaction, while also possibly being due to saturation of the metal coordination sphere by the presence of additional THF ligands. This is further implied when repeating the catalysis using **32** in THF, which resulted in a 3-fold decrease in rate along with only 50% conversion after 1 h.

**Scheme 12:**
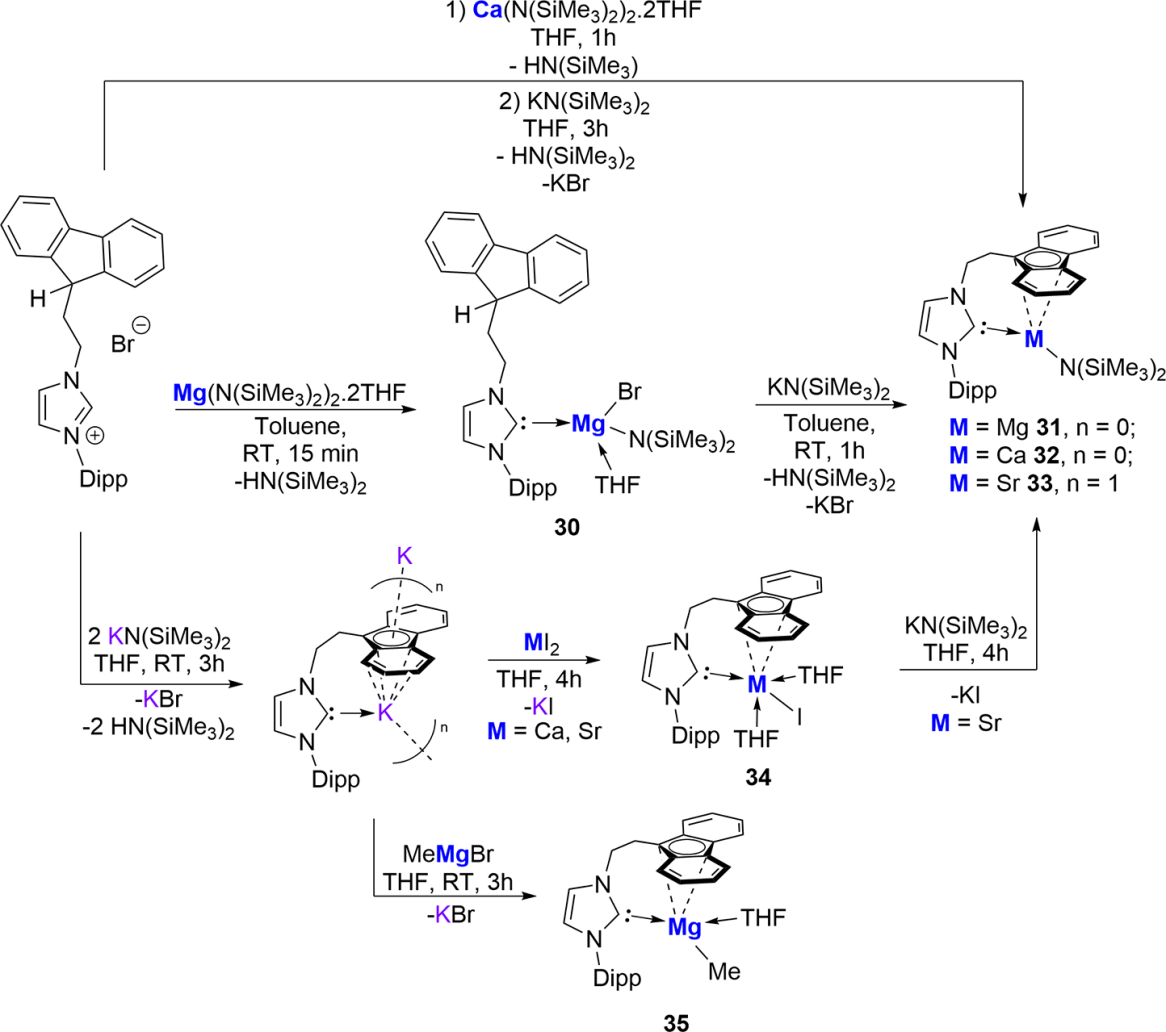
Mukherjee and Co-worker’s Reports on Fluorenyl-Tethered NHC-Mg, Ca, and Sr Complexes

A recent study by Trifonov and co-workers in 2023 introduced the dimeric, amido-functionalized NHC-calcium complex **36** (Figure 8) via addition of excess [Ca­(N­(SiMe_3_)_2_)_2_] to the imidazolium salt, which results in dearomatization of the pyridyl ring and formation of covalent M–N bonds.^73^ This was then screened as a potential precatalyst for both the hydrophosphination and hydroamination of styrenic-based monomers (Figure 8a,b). With catalyst loadings as low as 1 mol %, 99% conversion of styrene with diphenylphosphine to the anti-Markovnikov addition product was observed within 5 min at room temperature. Control of the formation of secondary and tertiary phosphine products could also be achieved using phenylphosphine through variation in the ratio of starting reagents. Furthermore, the use of **36** as a precatalyst for the intermolecular hydroamination of *p*-divinylbenzene with pyrrolidine and piperidine was explored, and again, similarly high conversions (>90%) with 5 mol % of **36** were achieved. Single and double addition of the aforementioned secondary amines could be obtained again by variations in the stoichiometries of the starting materials.

**Figure 8:**
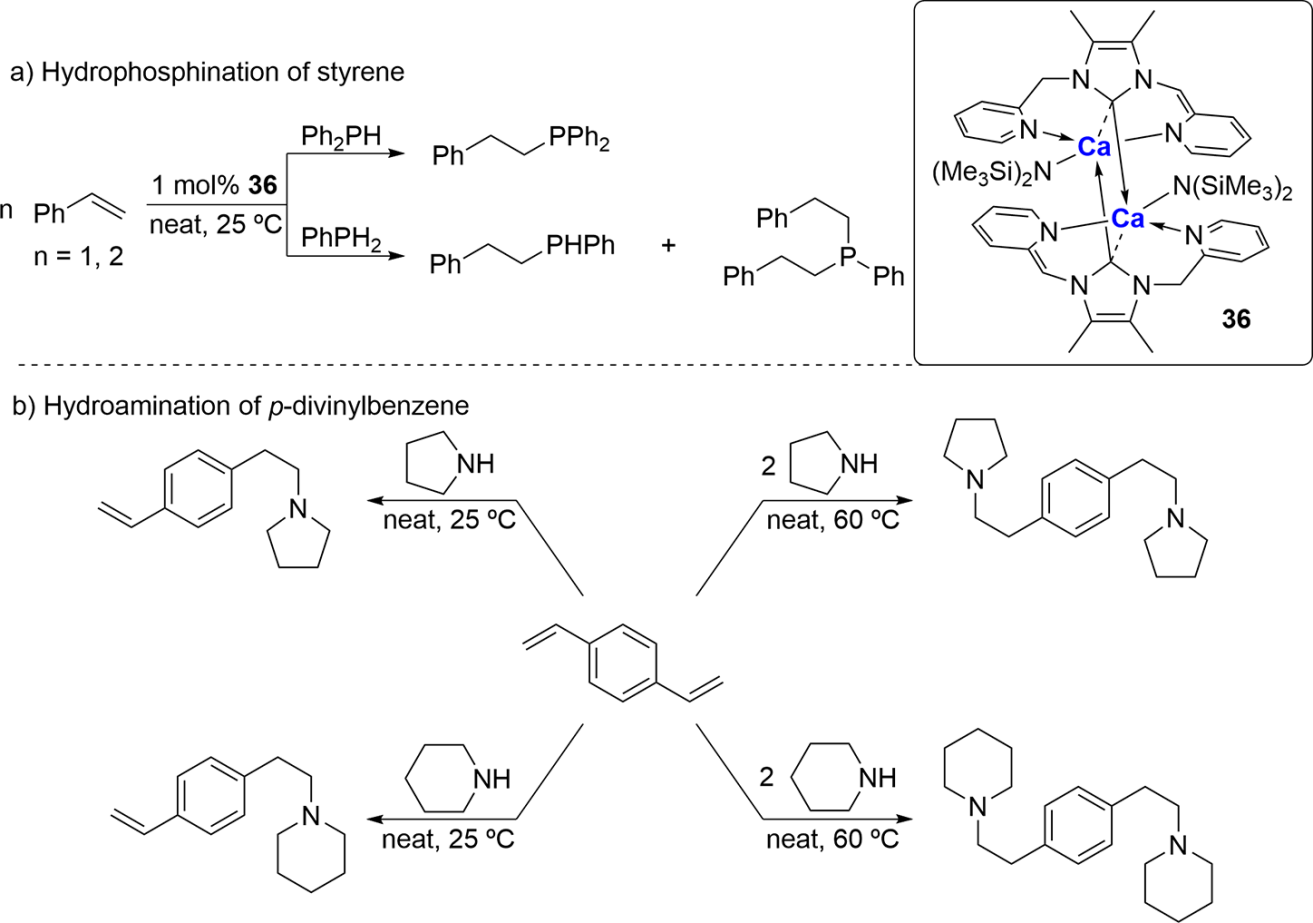
Amino-tethered NHC-Ca precatalyst for hydrophosphination and hydroamination of styrene and *p*-divinylbenzene, respectively.

### 3.4 Bis-NHC-Stabilized Alkaline Earth Complexes

Bis-NHC ligated complexes of the alkaline earth metals are similarly extremely rare and are predominantly used as ligand transfer agents. As such, only a select few intriguing examples are detailed herein.

The Westerhausen group in 2017 reported the synthesis of a series of heavy alkaline earth metal complexes stabilized by a pyridyl-bridged bis-NHC ligand, **37** (Scheme 13).^74^ While simple addition of the metal diiodide, for M = Sr and Ba, leads to the expected formation of the NHC-MI_2_ complexes **38b** and **38c**, addition of CaI_2_ to THF solutions of ligand **37** not only yielded the expected NHC-CaI_2_ complex **38a** but also enabled characterization of the separated ion pair complex **39**, in which coordination of two **37** ligands plus an additional THF molecule produces a 7-coordinate calcium cation. While no further spectroscopic data could be obtained for **39**, attempts to substitute one iodide anion for a noncoordinating BPh_4_^–^ anion afforded crystalline NHC-CaI_2_ complex **38a** in 37% yield along with an amorphous white solid believed to be the BPh_4_ dianion complex [(NHC)­Ca]^2+^[BPh_4_]^2–^ **40**. The authors suggest that a ligand scrambling process occurs, yielding **38a** and **40** from the target [(NHC)­CaI]^+^[BPh_4_]^−^ complex. Further efforts to synthesize soluble calcium ion pair complexes utilized the AlPh_4_ weakly coordinating anion, affording [(NHC)­CaI]^+^[AlPh_4_]^−^ **41** via treatment of **37** with [(THF)_5_CaI]^+^[AlPh_4_]^−^.

**Scheme 13:**
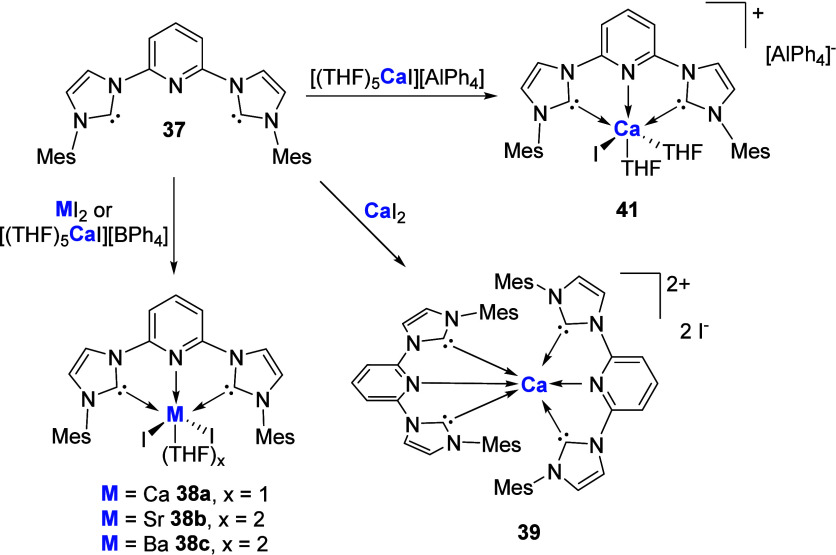
Synthesis of Pyridyl-Linked NHC Ca, Sr, and Ba Complexes

This was followed by research from the Munz group in 2019 who, using a similar pyridyl-linked NHC ligand, reported the Mg­(II) complexes **42a** and **42b** (Scheme 14) via initial *in situ* synthesis of the free carbene followed by complexation of MgBr_2_.^75^ There were no reported ion pair complexes in line with the previous Westerhausan report, with complexes **42a** and **42b** used solely as ligand transfer agents in the synthesis of Pd and Fe complexes.

**Scheme 14:**
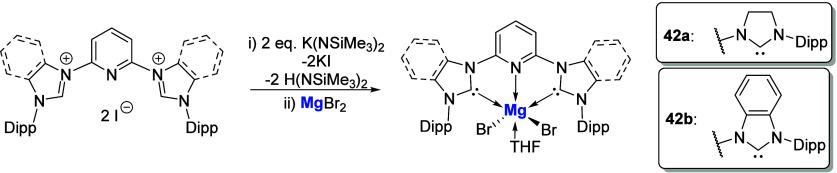
Synthesis of Pyridyl-Linked NHC-Mg Complexes

Building upon their previous work using fluorenyl-tethered NHCs, the Mukherjee group synthesized the dianionic bis-fluorenyl NHC-Ca­(II) complex **43** (Figure 9).^55^ Initial reaction formed the monoanionic species [NHC-Ca­(N­(SiMe_3_)_2_)_2_], with subsequent heating forming the expected dianionic complex **43**. No further reports utilizing this ligand have been forthcoming, although its potential for stabilization of element­(II) species is apparent.

**Figure 9:**
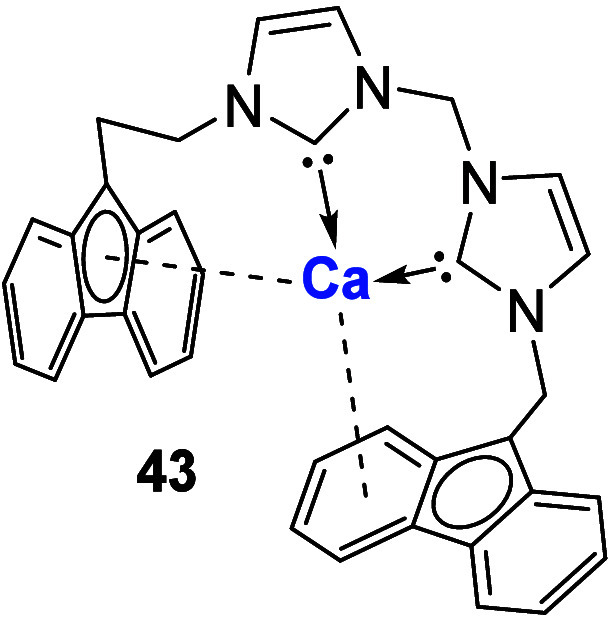
Bis-fluorenyl NHC Ca complex **43**.

## 4 NHCs in Group 13 Chemistry

NHCs have played a pivotal role in group 13 chemistry, particularly within stabilizing reactive species in their lower oxidation states. Within group 13, boron chemistry dominates the field in terms of the number of complexes isolated in unique coordination environments (i.e., multiple bonds,^76,77^ borylenes,^78^ boryl radicals^79,80^ ) and within catalytic application.^81–85^ As such, boron compounds will not be covered in this perspective article.

### 4.1 NHC-Stabilized Group 13 Complexes

NHC adducts of group 13 elements in their +3 oxidation state have been realized for all elements, from aluminum to thallium. However, due to the inert pair effect, the stability of the +3 oxidation state decreases upon descending the group, with the +1 oxidation state becoming more prevalent. This is nicely reflected in the comparison of the crystallographically characterized structures within the Cambridge Structural Database (CSD) for the series of NHCEX_3_ compounds, with a notable decrease in the number of entries on descending the group (Table 1). In addition to this, the only two examples of Tl­(III) NHC adducts are both the trichloride complexes,^86,87^ with no reported hydride or aryl/alkyl adducts.

**Table 1:**
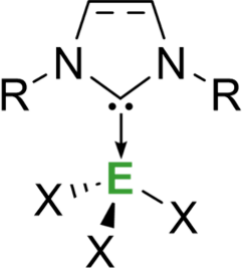
Reported Number of Structurally Characterized NHCEX_3_ Compounds

E	hydride	halide	aryl/alkyl	no. of CSD entries^a^
Al	√	√	√	125
Ga	√	√	√	65
In	√	√	√	31
Tl	X	√	X	2

a Also includes mixed hydride/halide/alkyl complexes.

One of the major uses of NHCs in group 13 chemistry is to stabilize compounds in their lower oxidation state. In 2010, Stasch and co-workers reported the use of their Mg­(I) reducing agent (Mg­(I) = [{(^Ar^NacNac)­Mg}_2_] (Ar = Dipp, Mes)) to access the first example of an *N*-heterocyclic carbene adduct of the parent dialane(4) via reduction of the Al­(III) hydride species using Mg­(I) (Scheme 15).^88^ More recently, our group has similarly investigated the use of both Mg­(I) and Al­(I) (Al­(I) = [(^Ar^NacNac)­Al] (Ar = Dipp)) as stoichiometric reducing agents toward various NHC-alanes whereby the choice of both reducing agent and NHC ligand influenced the reaction outcome (Scheme 15). Reactions with Mg­(I) dimers exclusively yielded the expected dialane complexes [{NHCAlH_2_}_2_] (NHC = IPr* **44**, IDipp **45**, ICy **46**; IPr* = 1,3-bis­(2,6-bis­(diphenylmethyl)-4-methylphenyl)­imidazol-2-ylidene), while use of Al­(I) gave rise to, depending on the steric demand of the NHC, an NHC-dialane, **44** (with IPr*); a cationic abnormal aluminum dihydride, **47** (with IDipp); or an asymmetric mixed-ligand dialane, **48** (with ICy).^89^ NHC-stabilized dialanes (both symmetric and asymmetric),^90^ digallanes, and diindanes^91,92^ have all been reported with the group 13 metal in its +2 oxidation state, although examples in the literature of their reactivity are limited.

**Scheme 15:**
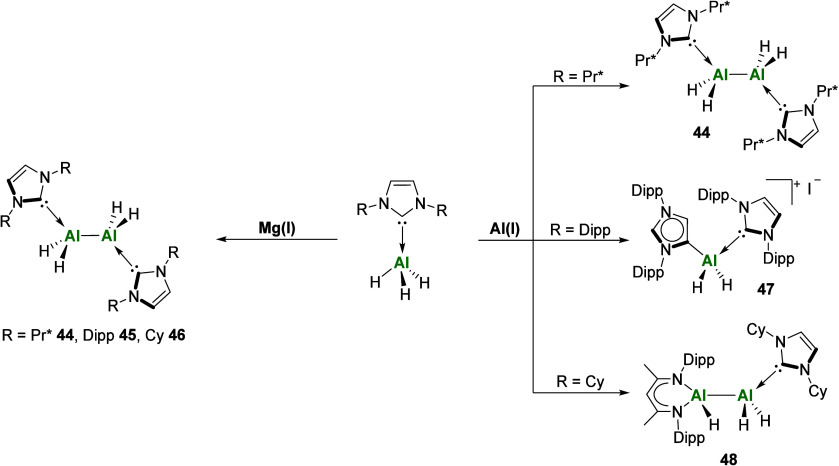
Divergent Reduction Chemistry of NHC-Alanes

NHC-aluminum complexes featuring aluminum in the +1 oxidation state were not attained until 2017, with Inoue and co-workers reporting the first example of a neutral dialumene, a compound featuring an aluminum–aluminum double bond (Scheme 16).^93^ Key to the isolation was the use of sterically demanding di-*ter*t-butyl­(methyl)-silyl (**49a**) groups and later the Tipp (**49b**) aryl group^94^ (Tipp = 2,4,6-triisopropylphenyl), which provided electronic and kinetic stabilization of the double bond, while the small ^Me^I*i*Pr NHC acted as an external electron donor. It is important to note the size of the NHC, as subsequent attempts by other groups have shown that following a similar synthetic protocol results in the formation of masked species (Scheme 16), i.e., reduction of NHCAl­(X)_2_R (X = Br or I, R = alkyl or aryl), where the NHC contains aryl wingtip substituents (e.g., IDipp) resulting in [2 + 4]-cycloaddition to yield a trialane^95^ (**50**) and use of the smaller IMe_4_ (IMe_4_ = 1,3,4,5-tetramethyl-imidazolin-2-ylidene) in combination with a larger aryl group resulted in C–H activation (**51**).^96^

**Scheme 16:**
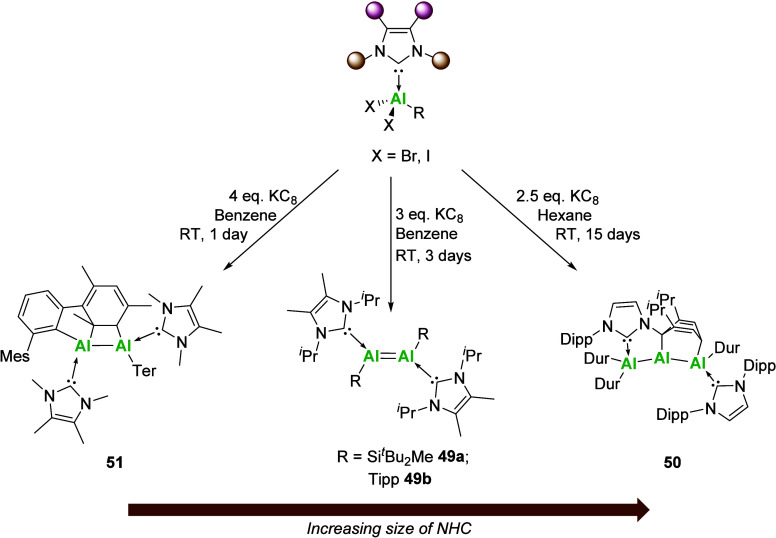
Reduction of NHCAl­(X)_2_R Complexes with Varying Size of NHC

Isolation of a neutral aluminum double bond requires a careful balance in sterics from both the NHC and ancillary ligand; however when isolated, it is a powerful tool for bond activations and catalysis. Formal [2 + 2]-cycloaddition of CO_2_ to compound **49a** results in the isolation of the CO_2_ fixation product (**52**), with retention of the Al–Al bond (Scheme 17). Heating compound **52** in the absence of CO_2_ results in cleavage of the C–O bond to yield a bridged carbonyl species (**53**). Meanwhile, in the presence of CO_2_, a six-membered carbonate species is formed (**54**). Further activation of N_2_O and O_2_ was also possible, producing the dioxo species (**55**) that could also be reacted with CO_2_ to yield **54.**

**Scheme 17:**
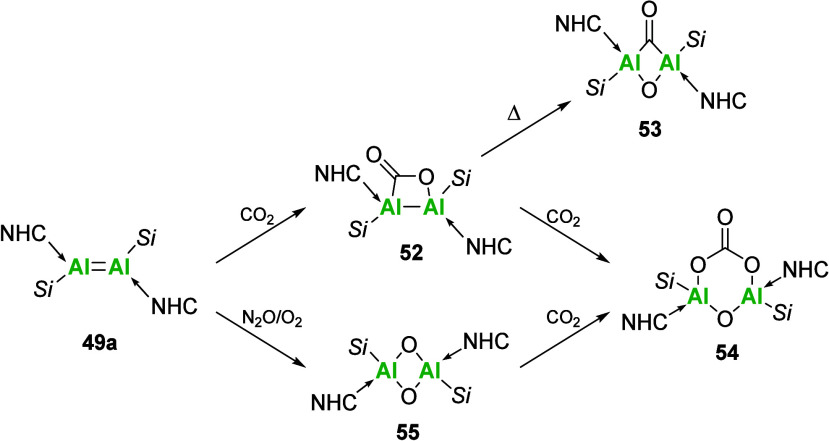
Stoichiometric Bond Activations of CO_2_, N_2_O, and O_2_ with Dialumene **49a** [Fn sch17-fn1]

a
*Si* = di-*tert*-butyl­(methyl)-silyl.

Importantly, dialumene **49a** was found to be a precatalyst in the catalytic reduction of CO_2_ (Scheme 18).^97^ **54** was found to be the active catalyst, and in the presence of HBpin, efficient turnover to the formic acid derivative was achieved. Mechanistic and computational studies revealed a facile transformation (−13.4 kcal mol^–1^ overall) proceeding via the initial coordination of HBpin to the exocyclic oxygen of the carbonate fragment followed by subsequent hydride transfer. This hydride transfer was proposed to be the rate-limiting step (−22.2 kcal mol^–1^), which was then offset by −26.6 kcal mol^–1^ upon the formation of the reduced carbonate (**56**). Finally, CO_2_ coordination on the opposite plane of the Al–Al bond led to the reformation of the carbonate moiety (**57**). The regeneration of **49a** was facilitated through the breakdown of **57** and subsequent release of the formic acid derivative as the CO_2_ reduction product.

**Scheme 18:**
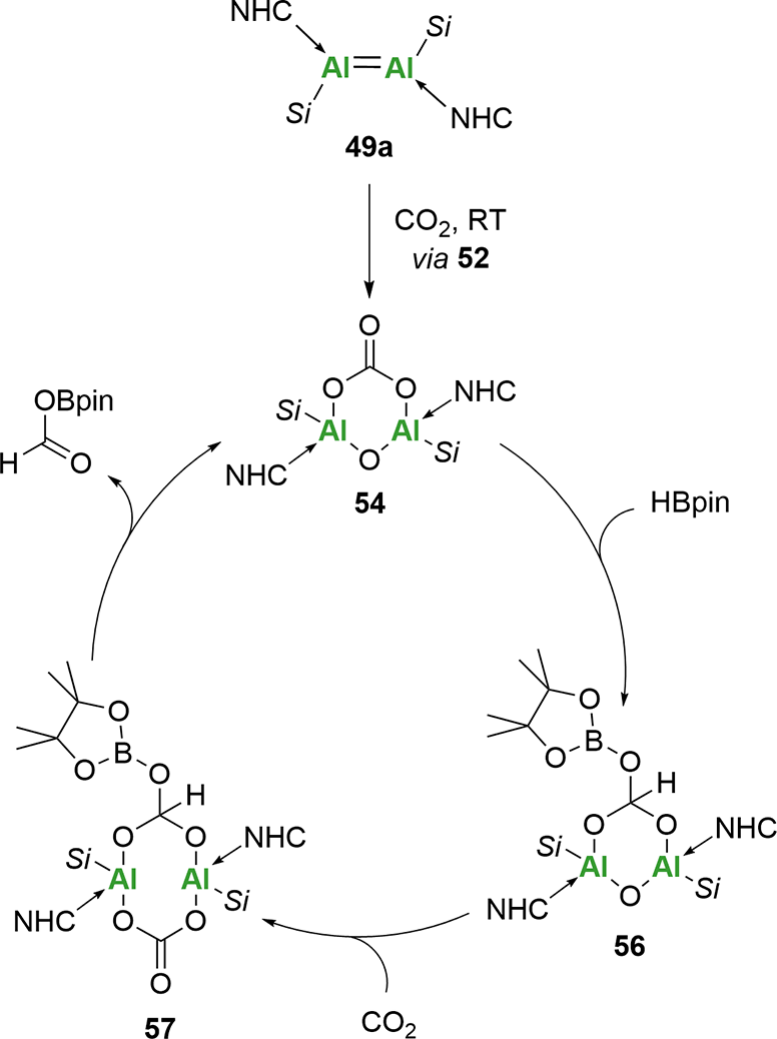
Proposed Catalytic Cycle for the Hydroboration of CO_2_ Using **49a** as a Precatalyst^a^

a NHC = ^Me^I*i*Pr. *Si* = di-*tert*-butyl­(methyl)-silyl.

Recently, the Inoue group expanded on the potential of **49a** as a precatalyst toward the 1,2-reduction of quinolines via hydride transfer from ammonia borane to selected quinoline substrates (Scheme 19), displaying excellent chemo- and regioselectivity and good functional group tolerance under mild conditions.^98^

**Scheme 19:**
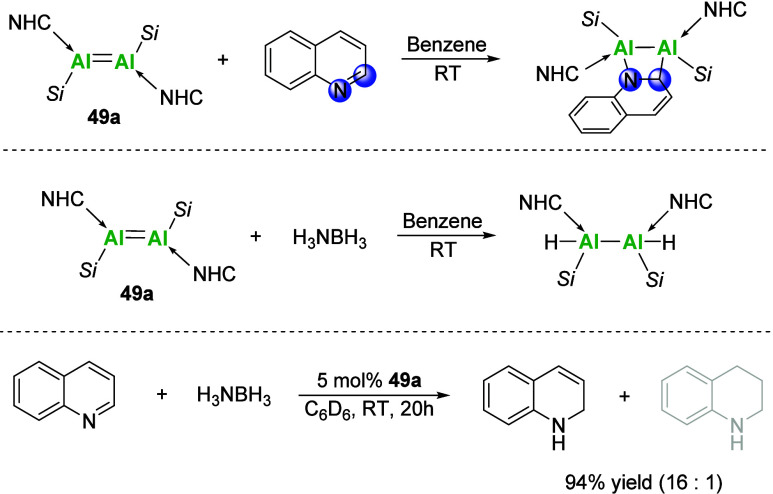
**49a** as a Precatalyst in the 1,2-Reduction of Quinoline^a^

a NHC = ^Me^I*i*Pr. *Si* = di-*tert*-butyl­(methyl)-silyl.

### 4.2 NHC-Stabilized Cationic Group 13 Complexes

A main feature of group 13 compounds is their high Lewis acidity, which typically decreases in strength upon descending the group, making boron the most potent Lewis acid in the group. This feature has long been exploited for catalytic application, with classic examples of Lewis acid catalysis in Friedel–Crafts^99^ and Ziegler–Natta^100^ polymerizations using aluminum. Further enhancement of the Lewis acid character can be achieved by removal of a substituent to access cationic complexes of the type [ER_2_]^+^ (E= group 13 element, R = monoanionic substituent, e.g., halide, alkyl, and aryl), thus providing an additional empty *p*-orbital and an increased charge on the group 13 center (Figure 10, type A). Complexes of this type are rare, as they require a combination of steric and electronic effects to provide stability to the highly reactive group 13 center

**Figure 10:**
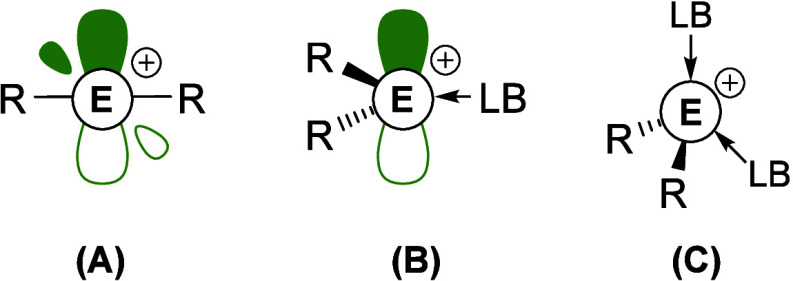
Classification of highly Lewis acidic group 13 cations.

A more common strategy for stabilization of highly Lewis acidic group 13 cations is to use Lewis bases, providing access to three-coordinate (type B) and four-coordinate (type C) cationic complexes. Lewis bases such as NHCs have paved the way in borenium (type B) and boronium (Type C) catalysis, showing enhanced reactivity over their neutral counterparts, and have been widely covered in several review articles.^83,101–107^ Advances in the heavier NHC-stabilized group 13 cationic complexes have been limited, but recent advances by Radius^108^ and Stephan^109^ have shown the versatility of these super Lewis acids. In 2023, Radius and co-workers reported the synthesis of [NHCAlMes_2_]^+^ cation **58** via hydride abstraction from the corresponding Al­(III) compounds. While **58** was shown to be a potent Lewis acid, via experimental and theoretical methods, it was also shown to exhibit FLP behavior. No interaction between **58** and PCy_3_ was observed spectroscopically, but upon addition of CO_2_, the insertion between the Al and PCy_3_ centers was observed to yield the CO_2_ fixation product **59** (Scheme 20).

**Scheme 20:**
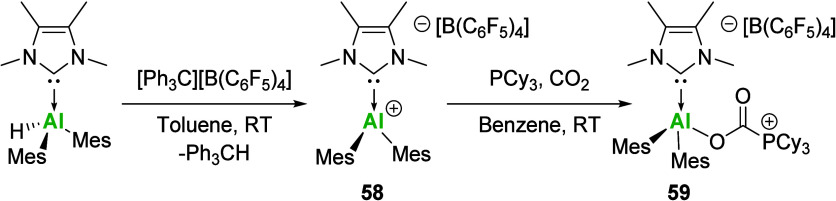
Synthesis and “FLP”-like Reactivity of Al­(II) Cations

Using the hydride abstraction method, Stephan and co-workers reported the synthesis and catalytic activity of **60** (Scheme 21). In their case, the reaction solvent (toluene) was found to coordinate to the Al center in a η^3^ fashion. Importantly, repetition of this reaction in fluorobenzene results in increased solubility of the cationic complex, along with coordination of the F atom of fluorobenzene to the Al center. The super Lewis acidity of **60** was shown by the ambient temperature fluoride abstraction from [SbF_5_]^−^ and in a series of organic transformations (Scheme 21). Low catalyst loadings of **60** enabled fast and near-quantitative conversion of 1-fluoroadamantane in hydrodefluorination and Friedel–Crafts reactions. Further applicability in the hydrosilylation of 2-norbornene was also demonstrated, highlighting the potential of NHC-stabilized aluminum cations in catalysis.

**Scheme 21:**
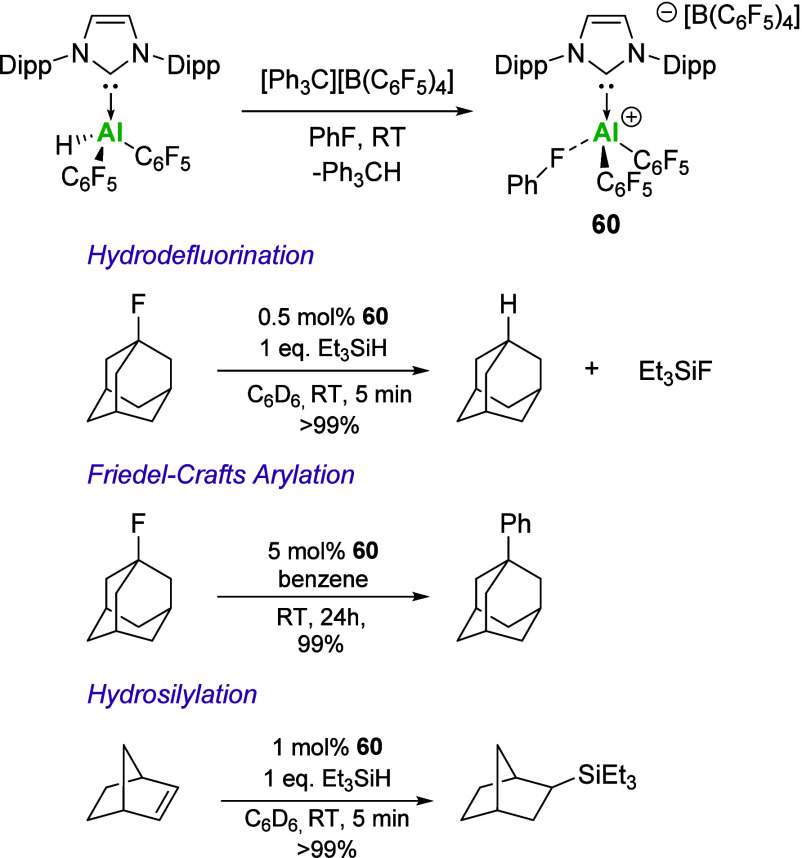
Synthesis of Super Lewis Acidic Cation **60** and Catalytic Application in Organic Transformations

### 4.3 Tethered-NHC Group 13 Complexes

As mentioned above, the combination of Lewis basic NHCs and Lewis acidic metals results in the formation of donor–acceptor-type complexes. While this has its advantages for stabilizing group 13 elements in unusual coordination modes, it can also result in metal–ligand cooperativity akin to FLP reactivity. This cooperativity, or hemilability, can be further exploited by use of anionic tethers as this allows for the dissociation of the NHC, but the tether will keep it within the vicinity of the metal center. In lanthanide chemistry, this has been used to great effect, with reversible addition/elimination reactions observed from the reaction of polar substrates across the metal–carbene bond.^11,110,111^

There is a growing interest in complexes of this type, particularly for aluminum, with several groups highlighting the non-innocence of the Al–NHC bond.^112–114^ Camp and co-workers reported the key influence of the choice of aluminum reagent on the resulting complex formation.^113^ Less sterically demanding groups resulted in the expected alkoxy-NHC complexes (Scheme 22; **61**), whereas larger groups showed preference for the formation of an imidazolium-aluminate zwitterion (**62**). It is of note that this trend continues down the group with alkylgallium reagents.^115^

**Scheme 22:**
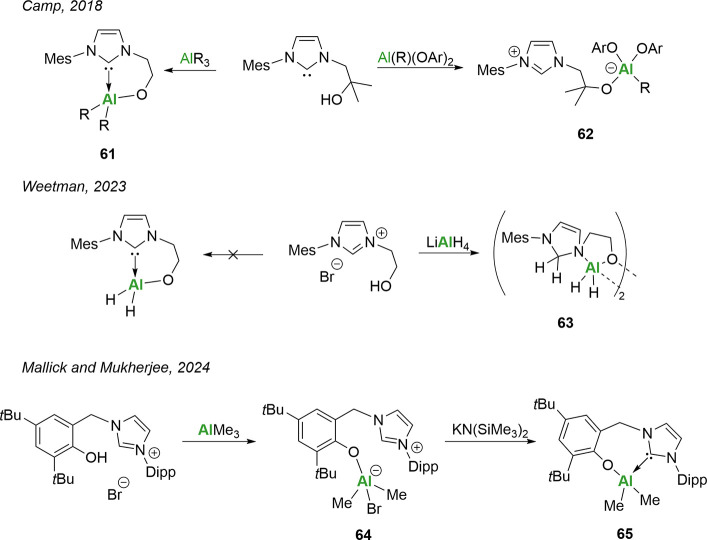
Non-innocence of Al-NHC Complexes

Further non-innocence was reported in 2023 by our group, wherein targeting the isolation of the alkoxy-NHC aluminum hydride species resulted in the loss of the carbene moiety and isolation of the *N*-heterocyclic aminal (Scheme 22). Hemilability was still observed in the complex but at the N–Al bond rather than the carbene–Al bond. Compound **63** was shown to act as an efficient hydride as it was able to reduce benzophenone and *N,N*′-dicyclohexylcarbodiimide. Importantly, its role in catalytic dehydrocoupling of amine-borane was investigated, achieving facile turnover in reduced time frames compared to other NHC or NHI-Al­(III) hydrides (NHI = *N*-heterocyclic imine). The improved catalytic performance is attributed to the increased ligand flexibility due to the hemilabile nature of the complex.

Mukherjee and co-workers also showed that aryloxides can be used as effective tethers and, in their case, were able to convert from the zwitterionic form (**64**) to the NHC adduct (**65**) (Scheme 22).^112^ Both of these complexes were used in the ring-opening polymerization (ROP) of ε-caprolactone (CL) and found to be inactive at room temperature; however, the NHC adduct (**65**) provided full conversion at 90 °C with a narrow dispersity (*Đ* = 1.10). Using benzyl alcohol (BnOH) as a cocatalyst with both **64** and **65** afforded room temperature catalysis, with the zwitterionic complex **64** proceeding at a surprisingly much faster rate. Mechanistic insights suggest that a zwitterion is the catalytically active species, as the addition of BnOH results in the protonation of the NHC–Al bond and formation of an Al-OBn containing zwitterion.

### 4.4 Bis-NHC Group 13 Complexes

Bis-carbene complexes of group 13 metals have scarcely been reported,^116–118^ with no new structurally characterized examples since 2014, providing plenty of opportunities for future development. Key examples again show the diverse coordination modes that are possible with this ligand class, with bimetallic formation preferred for the lighter elements and chelating for the heavier elements (Scheme 23).

**Scheme 23:**
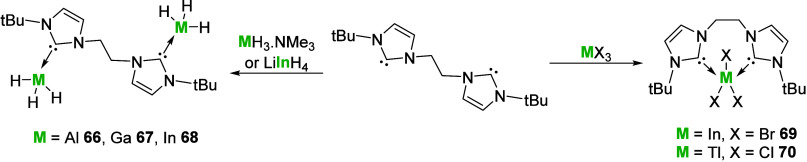
Bis-carbene Complexes of Group 13 Elements Showing the Diverse Coordination Modes

Use of the methylene linked bis-carbene enabled the isolation of a heterobimetallic aluminum–iron complex (**71**), where the aluminum center is formally in the +1 oxidation state.^118^ Attempts to isolate the Al­(I)-hydride via the reaction of **71** with KH or K­[BHR_3_] resulted in the isolation of **72a**–**b**, which arise from the deprotonation of the α-carbon position of THF reaction solvent, along with formation of dihydrogen (Scheme 24). The formation of the reactive intermediate **73** was implicated through additional reactivity studies toward C–O activation (**74**) and via isolation of the gallium analogue that was crystallographically characterized.

**Scheme 24:**
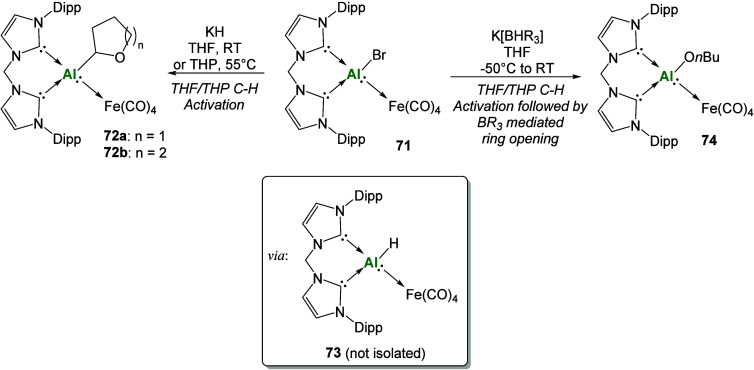
Synthesis and Reactivity of Al­(I) Heterobimetallic Bis-carbenes

## 5 Conclusions

While research and development into new NHC-ligated group 1, 2, and 13 metal complexes are currently receiving great interest across numerous research groups, with the first synthesis of such complexes dating back to the early 1990s, it is clear that there is still a long way to go before the full potential of these species is realized. This is particularly evident within the *s*-block metals, where only a select few examples of NHC-stabilized Mg and Ca complexes have been used in truly catalytic systems, e.g., hydroboration and hydrophosphination. For alkali metal NHC complexes, their uses in catalysis are nonexistent, and they are mainly used as ligand transfer agents. The majority of these complexes involve lithium, with only a select few examples of sodium and potassium species present in the literature. Prior to this year, the synthesis of NHC-Rb and Cs complexes had not even been reported, demonstrating the difficulty in stabilizing such species but also highlighting their untapped potential.

NHC usage has been prevalent in the stabilization and isolation of novel low-oxidation-state Al­(I) and Al­(II) species, including the isolation of the first example of an *N*-heterocyclic carbene adduct of the parent dialane(4) by Stasch and co-workers and the synthesis of the first Al=Al doubly bonded species by Inoue and co-workers. This in particular has been shown to enable both the stoichiometric and catalytic reduction of CO_2_. While rare, examples of cationic NHC aluminum­(II) species have been reported by the Radius and Stephan groups and subsequently shown to initiate hydrodefluorination and hydrosilylation due to their highly Lewis acidic nature, while also exhibiting FLP-like reactivity toward CO_2_. To date, only one example of a low-oxidation-state aluminum complex stabilized by either a tethered- or bis-NHC has been reported.

Looking forward, there is clearly great potential for expanding the catalytic applications of these NHC-main group species beyond simple hydroelementation reactions and toward classical redox-based systems dominated by transition metals, which would enable the utilization of earth-abundant metals and aid the development of greener, more sustainable chemical processes. While many challenges still exist, we anticipate that this area will continue to flourish and provide exciting advancements in the near future.
